# Variation of insulin‐related peptides accompanying the differentiation of *Aphis gossypii* biotypes and their expression profiles

**DOI:** 10.1002/ece3.10306

**Published:** 2023-07-15

**Authors:** Weili Jiang, Muhammad Nasir, Chenchen Zhao

**Affiliations:** ^1^ Basic Experimental Teaching Center of Life Sciences Yangzhou University Yangzhou China; ^2^ Agricultural Biotechnology Research Institute, Ayub Agricultural Research Institute (AARI) Faisalabad Pakistan; ^3^ Henan International Laboratory for Green Pest Control/College of Plant Protection Henan Agricultural University Zhengzhou China

**Keywords:** aphid, gene variation, genome, population evolution, RNA‐sequencing, transposon

## Abstract

Insulin signaling plays a critical role in regulating various aspects of insect biology, including development, reproduction, and the formation of wing polyphenism. This leads to differentiation among insect populations at different levels. The insulin family exhibits functional variation, resulting in diverse functional pathways. *Aphis gossypii* Glover, commonly known as the cotton‐melon aphid, is a highly adaptable aphid species that has evolved into multiple biotypes. To understand the genetic structure of the insulin family and its evolutionary diversification and expression patterns in *A. gossypii*, we conducted studies using genome annotation files and RNA‐sequencing data. Consequently, we identified 11 insulin receptor protein (*IRP*) genes in the genomes of the examined biotypes. Among these, eight *AgosIRPs* were dispersed across the X chromosome, while two were found in tandem on the A1 chromosome. Notably, *AgosIRP2* exhibited alternative splicing, resulting in the formation of two isoforms. The *AgosIRP* genes displayed a high degree of conservation between Hap1 and Hap3, although some variations were observed between their genomes. For instance, a transposon was present in the coding regions of *AgosIRP3* and *AgosIRP9* in the Hap3 genome but not in the Hap1 genome. RNA‐sequencing data revealed that four *AgosIRPs* were expressed ubiquitously across different morphs of *A. gossypii*, while others showed specific expression patterns in adult gynopara and adult males. Furthermore, the expression levels of most *AgosIRPs* decreased upon treatment with the pesticide acetamiprid. These findings demonstrate the evolutionary diversification of *AgosIRPs* between the genomes of the two biotypes and provide insights into their expression profiles across different morphs, developmental stages, and biotypes. Overall, this study contributes valuable information for investigating aphid genome evolution and the functions of insulin receptor proteins.

## Graphical abstract

Eleven IRPs were identified in *Aphis gossypii*. AgosIRP2 exists as an alternative splicing site. Most AgosIRP expression levels decreased when treated with pesticide.

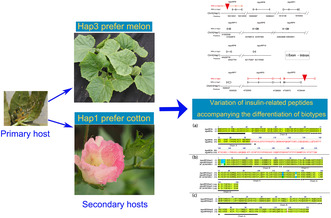

## 1 INTRODUCTION

*Aphis gossypii* Glover, commonly known as the cotton‐melon aphid, is a significant agricultural pest that causes substantial economic losses. It poses a threat by aggregating in large numbers to feed on plant sap and transmitting viral diseases to host plants (Blackman & Eastop, 2000). *A. gossypii* is highly polyphagous, capable of infesting over 600 plant species, including important crops like cotton and cucumber (Ebert & Cartwright, 1997). The ability of *A. gossypii* biotypes to adapt to different host plants is clearly distinct (Carletto et al., 2009; Zhang et al., 2018). In a study using the mitochondrial cytb gene region, researchers identified 57 haplotypes from 1046 *A. gossypii* individuals in northern China. Among these, Hap1 and Hap4 were the most prevalent biotypes, predominantly found on cotton plants, while Hap3 was the most common biotype associated with cucumber plants (Zhang et al., 2018).

*Aphis gossypii* exhibits a wide distribution across various geographical regions, and its ability to adapt to different host plants has contributed to its widespread prevalence. This adaptability is facilitated by its rapid evolutionary processes. *A. gossypii* has evolved into complex life cycles, reproductive strategies, host biotypes, wing polyphenism, and pesticide resistance to cope with different climates, hosts, and environments (Kwon & Kim, 2017; Loxdale & Balog, 2018; Shi et al., 2010; Wang et al., 2016; Zeng et al., 2021). Additionally, the body size and fertility of *A. gossypii* can be influenced by the nutritional conditions provided by the host plants (Nevo & Coll, 2001).

Hormones play a crucial role in mediating alternative phenotypes in insects (Nijhout, 1999). Juvenile hormones, ecdysteroid, and insulin (insulin‐related peptides, IRPs) are the three most important hormones (Ogawa & Miura, 2014; Simon et al., 2010). The insulin signaling pathway, which operates through a highly conserved signaling transduction pathway, is involved in determining and regulating various phenotypes in insects. In aphids, the insulin signaling pathway has been extensively investigated in *Acyrthosiphon pisum* for its role in wing dimorphism (Grantham et al., 2020; Shang et al., 2020), control of aphid life cycle during long‐day seasons (Barberà et al., 2019), and regulating embryo development (Guo et al., 2016). Additionally, the insulin signaling pathway has been reported as essential for the successful transition from nymph to the adult stage in *Aphis* (*Toxoptera*) *citricidus* (Ding et al., 2017).

Due to the functional diversity of the insulin signaling pathway, the number and structure of *IRPs* differ significantly between insect species. The number of *IRP* genes is ranged between 3 and 15 among aphids (Huygens et al., 2022; Nässel & Broeck, 2016). However, the functions of most aphid *IRPs* remain poorly characterized, as the annotation of *IRPs* in aphid genomes is still incomplete (Huybrechts et al., 2010). In a previous study using the *A. gossypii* genome (ASM401081v1), 10 *IRPs* were annotated (Huygens et al., 2022). Recently, chromosome‐level genome assemblies of two biotypes of *A. gossypii* were released (Zhang et al., 2022). By reannotating the IRPs in these two *A. gossypii* biotypes and utilizing RNA‐sequencing data, we aimed to investigate their genomic structures, gene variations, and transcriptional expression patterns. This analysis could provide new insights into the evolution and function of insulin signaling in aphids.

## 2 MATERIALS AND METHODS

### 2.1 *IRP* identification

A Hidden Markov Model (HMM) pattern analysis, based on the insulin protein family database (Pfam, PF00049 of insect), was used to identify the *IRPs* in Hap1 and Hap3 genome data of *A. gossypii* (Majoros et al., 2004; Zhang et al., 2022). Meanwhile, amino acid sequences of insect *IRPs* from GenBank (http://www.ncbi.nlm.nih.gov) were selected as template sequences and homology‐based searches were carried out in Hap1 and Hap3 genomes. Finally, putative *IRPs* of *A. gossypii* were manually corrected and compared to previously reported *IRP* sequences of the insect (Huygens et al., 2022).

### 2.2 Genome‐level variation analysis of *IRPs* between Hap1 and Hap3

Two methods were used to determine the position on the genome and to find the possible duplication of *AgosIRPs* genes; first, extraction of the position of all genes from the Hap1 and Hap3 genome annotation files were carried out (Zhang et al., 2022) and second, the putative positions of all genes were obtained by BLAST‐aligned *AgosIRPs* genes to the genome assemblies. Finally, the putative *AgosIRPs* position on the genomes was manually amended. To check the genome‐level variation of *AgosIRPs*, the gene fragments between upstream of gene stop and downstream of gene start were obtained from Hap1 and Hap3, respectively. Based on the data, the coding exon/intron structures were analyzed. In order to compare the *IRPs* genome sequences, deletions, insertions, and substitutions in Hap1 and Hap3 were carried out on MAFFT (version 7) with default parameters (Katoh et al., 2019). The CENSOR software tool was used to select the insect database to identify the type of transposon in the sequence (Kohany et al., 2006).

### 2.3 Insect samples and RNA‐sequences

Wingless individuals of *A. gossypii* were collected from cotton and cucumber plants sown in Jiangsu province, China (32.392° N, 119.422° E). The biotypes were determined as previously reported (Zhang et al., 2016, 2018), and single mother‐generated populations were obtained by rearing on the host plant leaves. Four populations of each biotype, Hap1, and Hap3, were chosen and reared on cotton and cucumber leaves under controlled conditions at 26 ± 1°C temperature, 65 ± 5% relative humidity with a photoperiod of 14 h light and 10 h dark. For RNA‐sequencing, one sample per population (Hap1 and Hap3 populations), each sample containing 50 wingless aphids of mixed ages, was selected. The whole body of the insect was used to extract total RNA, purified the mRNA for library generation, and RNA‐sequencing and raw reads were processed by FASTQC and Trimmomatic softwares (Bolger et al., 2014).

### 2.4 *IRP* gene structure and variation analysis

To determine the predicted transcripts from the genome and discover new transcripts, de novo assembly was performed by using TRINITY software (Grabherr et al., 2011). RNA‐sequencing data, including Hap1, Hap3, and 50 Sequence Read Archive (SRA) files, were downloaded from the NCBI public database. The SRA files correspond to four morphs of *an* adult aphid, *A. gossypii*, including alate parthenogenetic females, apterous parthenogenetic females, gynoparae, and male, different development stages of male and neonicotinoid insecticides treated samples (Table S1). Both mitochondrial *cytb* and *AgosIRPs* sequences were selected from de novo transcript sequences by homology searches using BLASTn. Mitochondrial *cytb* sequences were used to determine the biotypes of *A. gossypii*, according to a previous report (Zhang et al., 2018). Selected *AgosIRPs* sequences were manually adjusted for further structural analysis. The nucleic acid sequence and protein sequence of *AgosIRPs* were examined using the Vector NTI software. Alternative splicing of *AgosIRPs* was revealed by aligned RNA‐sequencing read to *AgosIRPs* coding region, using the trial version of Geneious software. Signal peptides were identified in all *AgosIRPs* using the SignalP prediction software. Potential cleavage sites of insulin were predicted at either specific single or pairs of basic residues of the general formula (R/K)–Xn–(R/K) (Seidah & Chrétien, 1997).

### 2.5 Phylogenetic tree construction

The *IRP* transcript sequences of aphids were searched using *AgosIRPs* in the NCBI website using both BLASTn and BLASTp tools. Whole genome annotation data of *Aphis glycines*, *Eriosoma lanigerum*, *Pentalonia nigronervosa*, and *Rhopalosiphum padi* were downloaded from AphidBase (https://bipaa.genouest.org/is/aphidbase/), the *IRP* transcript sequences were obtained performing local BLAST search. After manual processing, the transcript sequences of the *IRPs* were used to construct a phylogenetic tree using the PhyloSuite software (Zhang et al., 2020). Graphical representation of the tree was performed with iTOL (https://itol.embl.de/).

### 2.6 Expression analyses of *IRPs* in *A. gossypii*

Quantification of the *AgosIRP*s was carried out at the transcript level with Salmon (v0.12.0). The transcript per million (TPM) value was used to reads counts by normalizing the gene length first and then by stabilizing the sequencing depth. In this study, we analyzed the expression profile of *AgosIRPs* in several RNA‐sequencing libraries corresponding to different *A. gossypii* morphs (males, sexual females, parthenogenetic females, and winged females) are publicly available in the NCBI SRA database (Table S1). The expression cutoff of at least two fragments per library was mapped. SPSS (version 20.0) was used to evaluate the differences in TPM values. To analyze the differences between the data with normal distribution, ANOVA was performed, while to analyze the data without normal distribution, the Mann–Whitney U test was carried out.

## 3 RESULTS AND DISCUSSION

### 3.1 *IRPs* identification in *A. gossypii*

To date, there has been limited in‐depth research on *IRPs* in *A. pisum*. The exact numbers o*f IRPs* in other aphid species have not been conclusively reported yet (Cuti et al., 2021; Gronke et al., 2010; Guo et al., 2016; Huygens et al., 2022; Shang et al., 2012). In this study, we identified a total of 11 insulin‐related peptide genes (*AgosIRP1*‐*AgosIRP11*) in two biotypes of *A.gossypii*. Among these 11 *AgosIRPs*, 10 genes were already present in the NCBI‐annotated database, which were based on the genomes previously published by our research group (Zhang et al., 2022). *AgosIRP*11 was a novel *IRP* identified in *A. gossypii*, belonging to the insulin‐like growth factor superfamily. The *IRPs* number varied from 3 to 15 among aphids, and this variability could be attributed to the division of aphid species and the limited availability of comprehensive genomic information for all aphid species. Different aphid species may possess different numbers of *IRPs* due to evolutionary adaptations and species‐specific requirements (Huygens et al., 2022). Genomic data have proven to be valuable in improving the annotation of insect genes, including those encoding *IRPs*. By comparing the *IRP* gene sequences obtained from annotated genomes, such as those of *A. pisum*, *Myzus persicae*, and *Rhopalosiphum maidis*, to the sequences in the NCBI database, significant alignments were observed. These alignments were mostly generated from high‐quality genome assemblies. However, to determine the exact number of *IRPs* in each aphid species, further investigations are necessary, particularly through the analysis of aphid genomic databases. Continued research and exploration of aphid genomes will provide valuable insights into the diversity and characteristics of *IRPs* in different aphid species.

### 3.2 *IRPs* in Hap1 and Hap3 *A. gossypii* genomes

**FIGURE 1:**
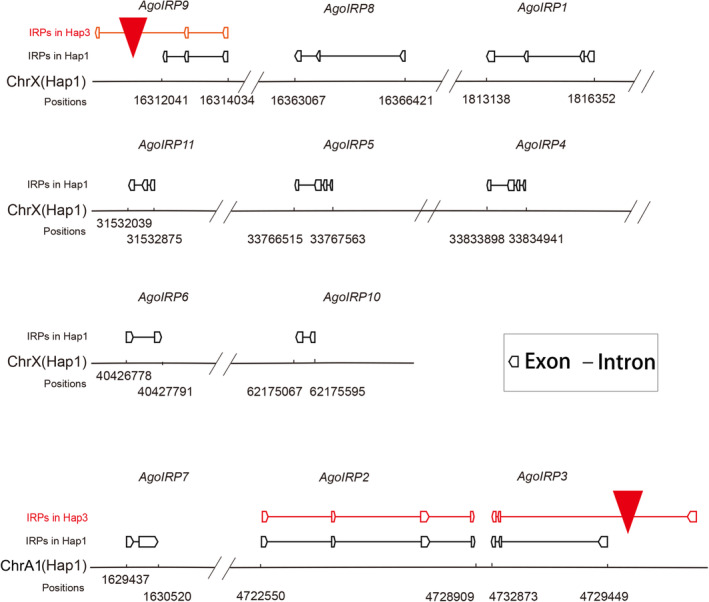
Schematic of the *AgosIRPs* located on *Aphis gossypii* chromosomes.

In *A. gossypii*, the species under investigation, the genome consists of four chromosomes, including one sex chromosome (X) and three autosomes. Among the insulin‐related peptide (*AgosIRP*) genes, eight genes were found scattered across the X chromosome. The distance between *AgosIRP5* and *AgosIRP6* on the X chromosome was found to be the shortest, measuring 65 kb. On the other hand, the remaining three *AgosIRPs* were located on the A1 chromosome, with two of them arranged in a tandem fashion (Figure 1). Interestingly, in aphids, it appears that majority of *IRP* genes are situated on the X chromosome. For instance, in the case of *A. pisum*, four *ApisIRPs* were located on the X chromosome, while three *ApisIRPs* were found on autosomes (Huygens et al., 2022). However, the distribution pattern of *IRP* genes differs in other insect species, such as Diptera. For example, in Anopheles gambiae, a species of mosquito, out of the seven insulin‐like peptide genes, five were located on autosomes, while two were found on the X chromosome (Krieger et al., 2004). Similarly, in *D. melanogaster*, five DILP genes were situated on autosomes, and two were present on the X chromosome. The region surrounding the DILP genes in Drosophila has provided evidence of ongoing adaptive processes, as suggested by patterns of genetic variation in proximity to these genes (Guirao‐Rico & Aguade, 2013).

The comparison of coding and non‐coding regions can provide insights into the reported differences in host ranges and life cycle diversity between Hap1 and Hap3 in *A. gossypii* (Andolfatto, 2005). The coding exon number in *AgosIRPs* were ranged from 2 to 4; however, no variation in coding exon numbers were observed among Hap1 and Hap3. The length of the coding regions varied, with the longest region measuring 3.4 kb and the shortest region measuring 0.5 kb. Four *AgosIRP* coding regions were conserved between Hap1 and Hap3, with only one having single‐nucleotide polymorphism (SNP) in each gene. However, two *AgosIRP* coding regions, *AgosIRP3* and *AgosIRP9*, showed difference of more than 2 kb length between Hap1 and Hap3. Further analysis showed that that these regions contained transposon insertions, specifically the piggyBac transposon in Agos*IRP3* and the SINE2 transposon in Agos*IRP9* (Figure 1).

It should be noted that changes in the number of repetitive DNA elements, including transposons, contribute to the variation in genome size between Hap1 and Hap3 (Zhang et al., 2022). Transposons are repetitive DNA elements capable of moving within the genome and are known to play a significant role in insect adaptation. For example, transposon insertions in the genome of *M. persicae* have been associated with potent insecticide resistance (Panini et al., 2021). However, the specific functions of the transposons inserted in the coding regions of *AgosIRPs* remain unidentified. It is worth mentioning that the piggyBac transposon system is widely used for genetic manipulation in insects and has been reported in *A. gossypii*, with nine piggyBac transposons identified in a previous study (Luo et al., 2011; Yusa, 2015). These transposons exhibit high sequence similarity with each other.

### 3.3 *IRPs* diversity among aphids

**FIGURE 2:**
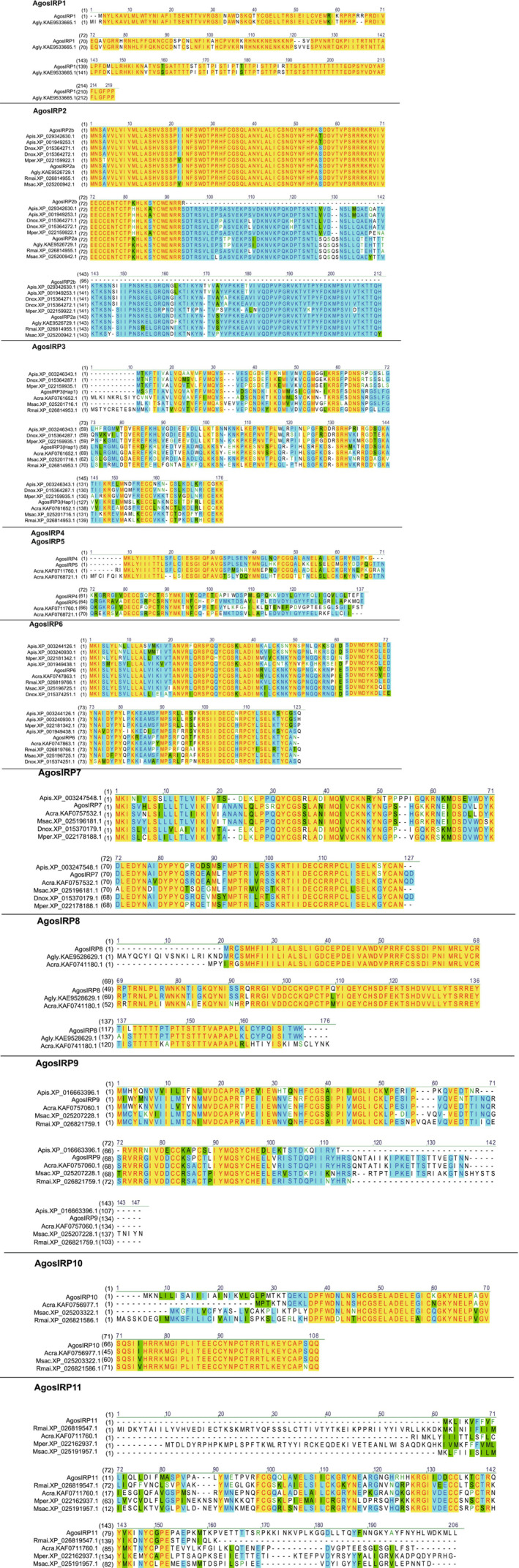
Predicted structure and gene variation of IRPs between aphids. Sequence names are indicated by a prefix formed from the abbreviated species name (*Aphis craccivora*, Acra; *Aphis glycines*, Agly; *Acyrthosiphon pisum*, Apis; Daktulosphaira vitifoliae, Dv; *Diuraphis noxia*, Dnox; *Melanaphis sacchari*, Msac; *Myzus persicae*, Mper; and *Rhopalosiphum maidis*, Rmai), followed by the IRP protein sequences accession number.

**FIGURE 3:**
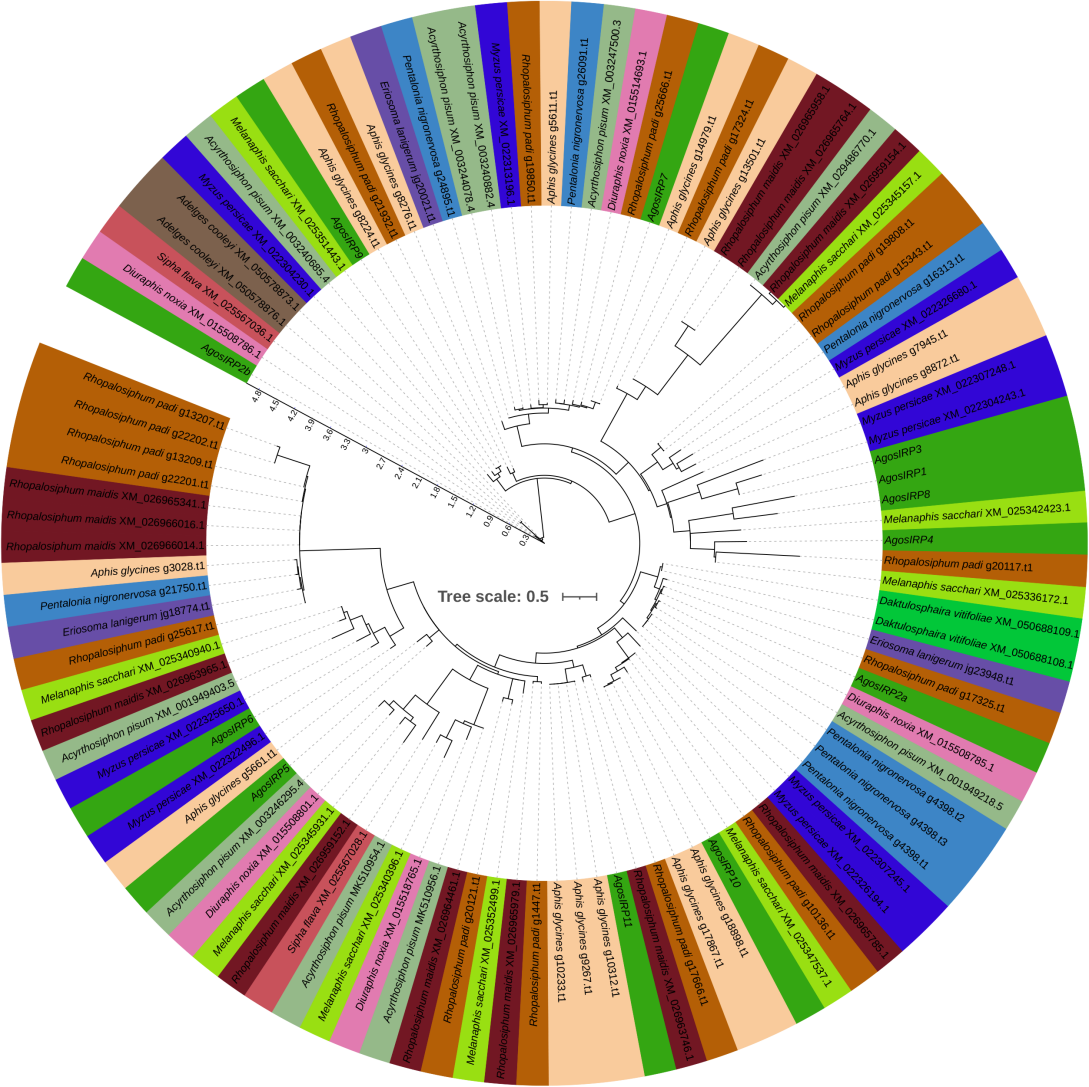
Phylogenetic tree based on nuclear acid sequences of *IRP* transcripts from various aphid species. *IRP* transcripts sequence was aligned with MAFFT v7.505 using “‐‐auto” strategy and codon alignment mode. Gap sites were removed with trimAI v1.2rev57 using “‐automated1” command. ModelFinder v2.2.0 was used to select the best‐fit model using BIC criterion. Maximum likelihood phylogenies were inferred using IQ‐TREE v2.2.0 under the model automatically selected by IQ‐TREE (“Auto” option in IQ‐TREE) for 20,000 ultrafast bootstraps, as well as the Shimodaira–Hasegawa–like approximate likelihood‐ratio test. The IRPs from different species are highlighted by various colors. The labels include the species name and the corresponding *IRP* transcript accession number.

In our study, we performed a comparison of *IRPs* in aphids to investigate gene evolution. We utilized aligned amino acid sequences and constructed a phylogenetic tree based on nucleic acid sequences (Figures 2 and 3). Among the *IRPs* identified in *A gossypii*, AgosIRP6 and AgosIRP7 represented two typical insulin‐like peptides. Similarly, in pea aphids, four *IRPs* were found, while other aphid species from different genera also exhibited two typical insulin‐like peptides, except for *R. maidis*, which had only one typical insulin‐like peptide (Huygens et al., 2022). The conservation of these two IRPs across aphids was evident, with an overall minimum range of 74% amino acid identity.

Furthermore, we identified homologous amino acid sequences of AgosIRP3 in eight other aphid species. All of these genes exhibited a characteristic pattern of 12 amino acid residues between two cysteine residues in the B chain, but variations were observed in the A chain. *A. gossypii*, *M. persicae*, and *Aphis craccivora* possessed an additional residue in the A chain, resulting in a CC(X)4C(X)8C motif (Figure 2). This motif has also been observed in DILPs of *Drosophila melanogaster* and ILPs of mosquitoes but differs from the IRPs found in aphids. It is worth noting that these genes with 11 amino acid residues between two cysteine residues in the B chain have been implicated in various physiological processes such as adult fat body function, lifespan extension, and determination of adult body size in previous studies (Bai et al., 2012; Okamoto et al., 2009; Sharma et al., 2019).

AgosIRP2 and AgosIRP8 belong to the highly conserved IRP family, with a higher amino acid identity (>88% and >84%, respectively). The AgosIRP2 family contains seven aphid species, while the *AgosIRP8 f*amily contains three aphid species belonging to the Aphis genus. Interestingly, the *AgosIRP2* family genes were located on two distinct branches of phylogenetic tree. Among seven aphids, ApisIRP6 showed >74% full lengths amino acid identity. *AgosIRP*9 represents another gene that exhibits a structural similarity to insulin‐like growth factors, showing >62% amino acid identity across its full length among the five aphid IRPs (Figures 2 and 3).

On the other hand, AgosIRP4, AgosIRP5, and AgosIRP11 display an additional residue in the A chain, resulting in a CC(X)_3_C(X)_9_C motif (Figure 2). The AgosIRP10 homologous genes are conserved among four aphid species, with almost identical amino acid sequences at the C‐terminal. In contrast, the AgosIRP4 and AgosIRP5 homologous genes are conserved across aphid species at the N‐terminal, suggesting that these genes may have evolved, diverged, and duplicated from a common ancestor (Figure 2; Irwin, 2021).

### 3.4 *IRPs* diversity between two biotypes *A. gossypii*

**FIGURE 4:**
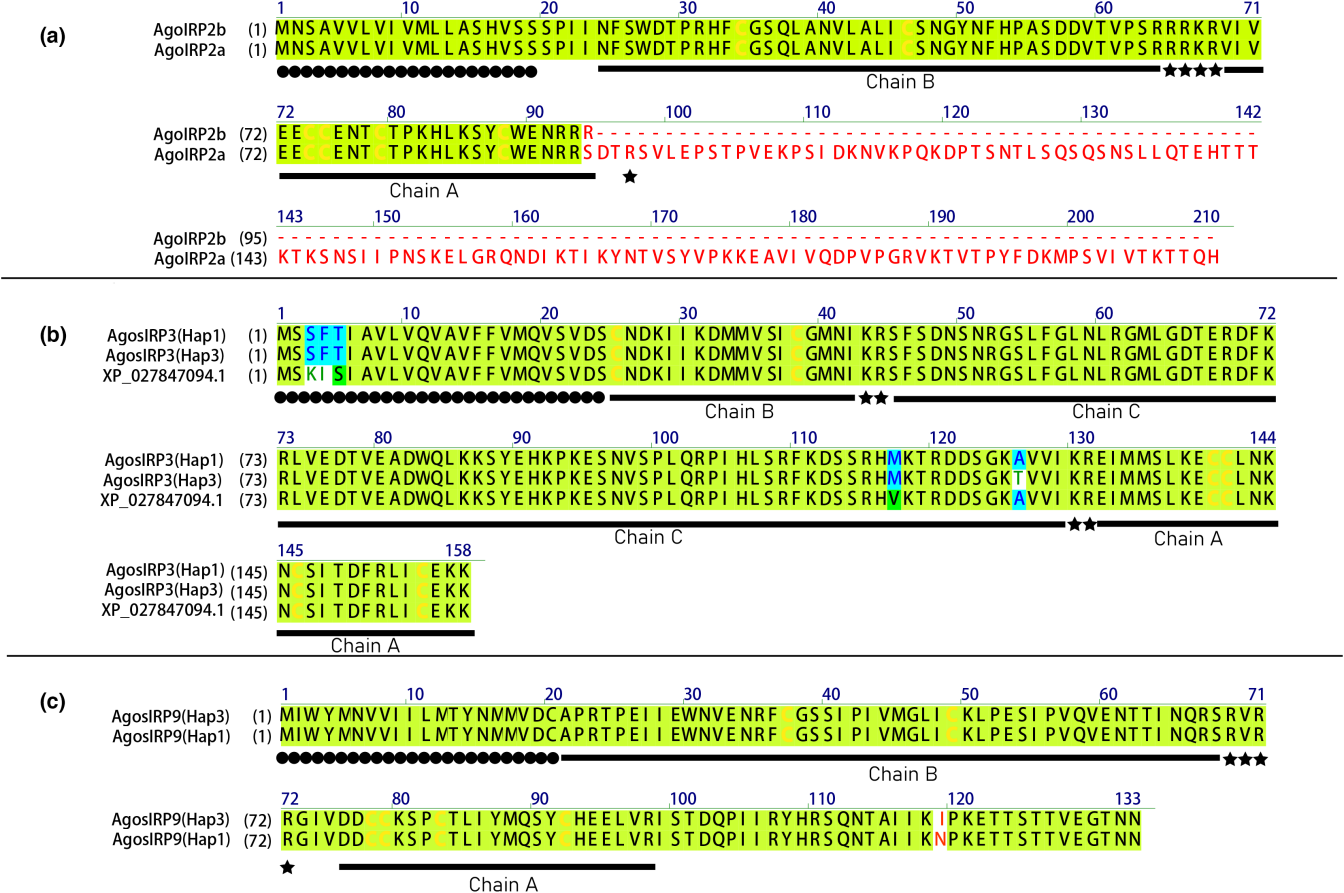
Predicted structure and gene variation of AgosIRPs between Hap1 and Hap3. (a) AgosIRP2 exists in one alternative splicing site in ORF region, and is present in two isoforms (AgosIRP2a and AgosIRP2b); amino acid different between Hap1 and Hap3 of AgosIRP3 (b) and AgosIRP9 (c). Disulfide bonds between conserved cysteines are indicated by yellow color; the black dot below the sequence indicates the signal peptide; the solid black line below the sequence indicates the A chain or B chain; and black five‐pointed star denote canonical prohormone convertase or furin cleavage sites.

The divergence between Hap1 and Hap3 of *A. gossypii* occurred approximately 15.72 M.Y.B.P. (Zhang et al., 2022). Despite this divergence, the *IRPs* in these two biotypes remain highly conserved. Only three *IRPs* have been identified as exhibiting diversity, and among them, *AgosIRP3* and *AgosIRP9* have a single SNP each, resulting in one amino acid difference between Hap1 and Hap3, respectively. Notably, these variant amino acids are not located on any functional chain (Figure 4). It is worth mentioning that variations in *IRP* genes within species are rarely reported. However, in *Anopheles gambiae*, a SNP was detected in the gene encoding insulin‐like peptide, which was suggested to be associated with parasite infection (Horton et al., 2010).

**FIGURE 5:**
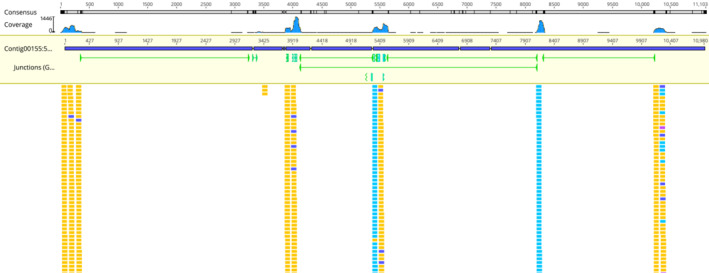
Mapped RNA‐seq reads were used to show novel mRNA alternative splicing in AgosIRP2.

In insects, alternative splicing is a common feature that leads to the creation of multiple protein isoforms, which increases diversity of proteome (Zhao et al., 2021). Insulin signaling pathway regulates several genes through alternative mRNA splicing, even the insulin receptor has known splice variants (Västermark et al., 2013). Sexual differentiation in the *Bombyx mori* is regulated by sex‐specific splicing of the protein that binds to the mRNA of insulin‐like growth factor II (Suzuki et al., 2014). In this study, we reported a novel mRNA alternative splicing of *A. gossypii*. Specifically, *AgosIRP2* was found to undergo alternative splicing, resulting in two isoforms: *AgosIRP2a* and *AgosIRP2b* (Figures 4 and 5). This splice variant resulted in the short isoform, *AgosIRP*2b, because it lacks the third coding exons and creates mRNA with premature stop codon at beginning of fourth coding exons. *AgosIRP*2 showed features similar to insulin‐like growth factor, and the portion that is absent, corresponds to entire E peptides (Figure 4). In vertebrates such as humans, mice, and sheep, insulin‐like growth factor genes undergo alternative splicing to produce multiple isoforms, and different transcripts contain distinct E peptides in these isoforms (Dai et al., 2010; Song et al., 2021). However, complete absence of the entire E peptide region, as observed in *AgosIRP2b*, has not been reported in insect‐based *IRPs* before. The E peptides may function either alone or in conjunction with mature insulin‐like growth factor (Brisson & Barton, 2012; Hede et al., 2012). Further studies are required to investigate the biological role of E peptides in *A. gossypii*.

### 3.5 Expression profile of *IRPs* in polymorphism *A. gossypii*

It is observed that *A. gossypii* primarily reproduces parthenogenetically for most of the year, with alate or apterous parthenogenetic females being produced. However, in the fall, *A. gossypii* is capable to produce alate gynoparae and males. The gynoparae migrate to primary host plants, where they give rise to sexual females that mate with alate males. The mated females then lay fertilized eggs, which undergo overwintering (Kwon & Kim, 2017; Liu et al., 2014; Miura et al., 2003). In the case of *A. pisum*, both sexual females and males are produced by gynoparae (Miura et al., 2003). Based on RNA‐sequencing data, it was found that four *AgosIRPs* (*AgosIRP2*, *AgosIRP3*, *AgosIRP6*, and *AgosIRP7*) are ubiquitously expressed in different morphs of *A. gossypii*. All four genes are located on the A1 chromosome, except for *AgosIRP2*, which is located on the X chromosome. These four *AgosIRPs* show uniform and high expression values, except for *AgosIRP7*, which exhibits the highest expression in adult gynoparae. Interestingly, the remaining seven *AgosIRPs* show specific expression patterns, being expressed only in adult males with low TPM values (Table 1). It is important to note that most of the RNA‐sequencing libraries used in this study were constructed using adult aphids. However, since *AgosIRPs* may play specific roles in different developmental stages, tissues, or cells, further studies are needed with additional samples from various developmental stages and tissues to confirm the expression patterns of *IRPs* in *A. gossypii*.

**TABLE 1:** Expression profile of *AgosIRPs* in *Aphis gossypii* polymorphism.

Genes	Wing parthenogenetical adult	Wingless parthenogenetical adult	Gynopara adult	Male	Sexual female adult
AgosIRP1	ND	ND	++	+	ND
AgosIRP2	+++	++++	++++	+++	++++
AgosIRP3	+++	+++	++++	+++	+++
AgosIRP4	ND	ND	++	++	ND
AgosIRP5	ND	ND	++	++	ND
AgosIRP 6	++	++	+++	+++	++
AgosIRP7	+	+	++	+	+
AgosIRP8	ND	ND	++	++	ND
AgosIRP9	ND	ND	++	+	ND
AgosIRP10	ND	ND	++	+	ND
AgosIRP11	ND	ND	+	+	ND

*Note*: Sequence Read Archive (SRA) files were downloaded from the NCBI public database, more than three SRA files in each polymorphism except sexual female adult, which only have one SRA file. +, TPM (transcript per million) value <1; ++, 1 ≤ TPM value <50; +++, 50 ≤ TPM value <100; ++++, 100 ≤ TPM value.

Abbreviation: ND, not determined.

### 3.6 Gene structure and expression patterns of *IRPs* in *A. gossypii*

In this study, the expression levels of *AgosIRPs* were analyzed in four morphs of adult aphid (alate parthenogenetic females, apterous parthenogenetic females, gynoparae, and males), different development stages of male, two biotypes (Hap1 and Hap3) and neonicotinoid insecticides treated samples (Hirata et al., 2017). The RNA‐sequencing data were analyzed with Kallisto, mostly used to align more distinct transcripts with very low TPM values (Ajaykumar & Yang, 2022). *A. gossypii* was found to contain four *AgosIRPs* that are ubiquitously expressed. Among these, *AgosIRP6* and *AgosIRP7* show structural similarities to genes that produce insulin, *AgosIRP2* shows structural similarities to insulin‐like growth factors, and *AgosIRP3* is a structurally divergent IRP similar to *ApisIRP11* (Huygens et al., 2022).

**FIGURE 6:**
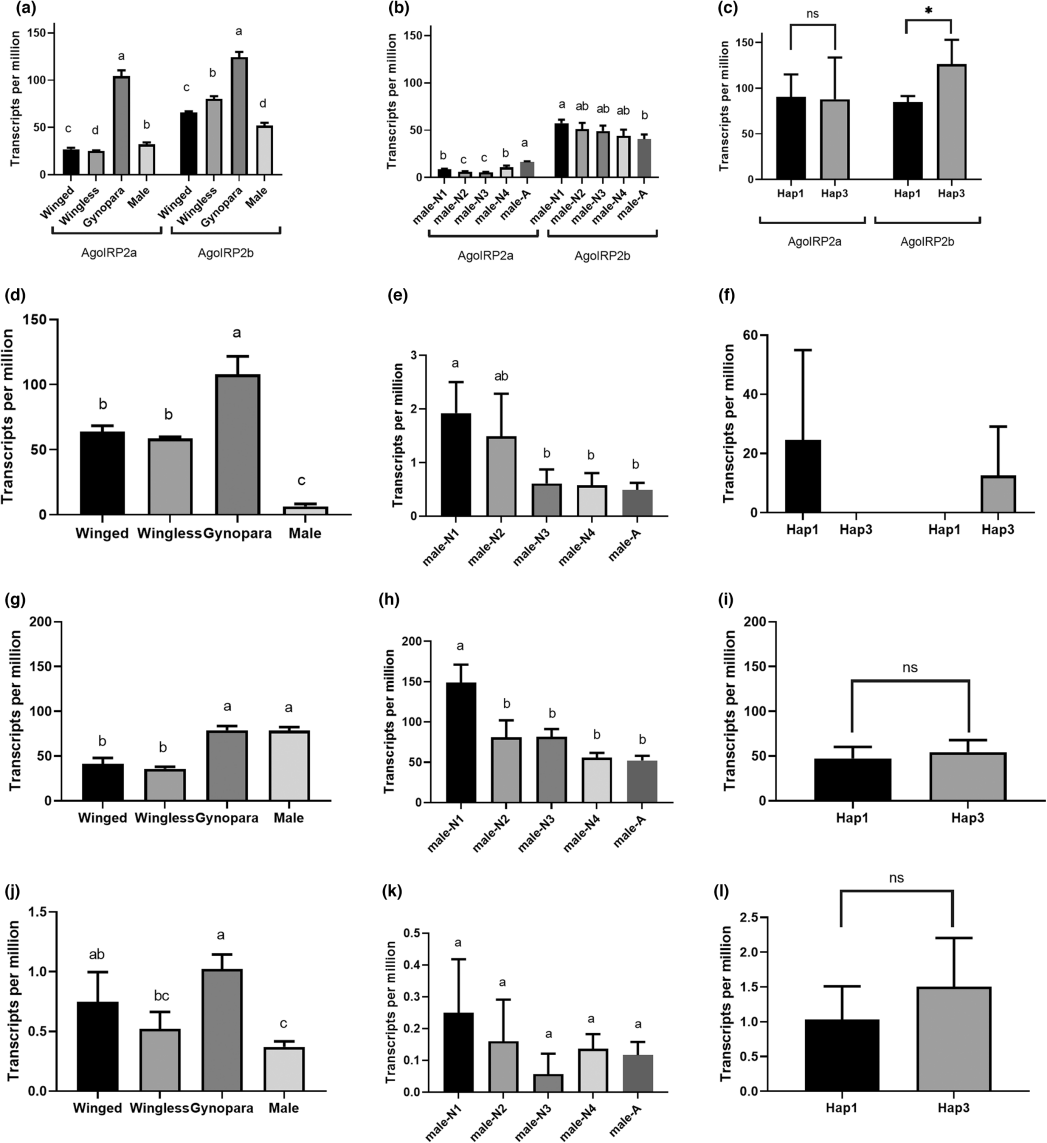
Relative expression quantity of *AgosIRPs* in polymorphism *Aphis gossypii*. *AgosIRP2* in four morphs of *A. gossypii* adult aphid (including alate parthenogenetic females, apterous parthenogenetic females, gynoparae, and male) (a), different development stages of male (b), biotypes Hap1 and Hap3 (c); *AgosIRP3* in four morphs of *A. gossypii* adult aphid (including alate parthenogenetic females, apterous parthenogenetic females, gynoparae, and male) (d), different development stages of male (e), biotypes Hap1 and Hap3 (f); *AgosIRP6* in four morphs of *A. gossypii* adult aphid (including alate parthenogenetic females, apterous parthenogenetic females, gynoparae, and male) (g), different development stages of male (h), biotypes Hap1 and Hap3 (i); *AgosIRP7* in four morphs of *A. gossypii* adult aphid (including alate parthenogenetic females, apterous parthenogenetic females, gynoparae, and male) (j), different development stages of male (k), biotypes Hap1 and Hap3 (l). First instar nymph male (male‐N1), second instar nymph male (male‐N2), third instar nymph male (male‐N3), fourth instar nymph male (male‐N4), and adult male (male‐A). The *Y*‐axis was the transcript per million (TPM) value (mean ± standard error of the mean). Different lowercase letters (a–d) indicate significant differences between treatments (*p* < .05) according to the post hoc Tukey's HSD method; *p < .05, ns, no significant difference, data were analyzed by Mann–Whitney *U* test.

Two spliced isoforms, *AgosIRP2a* and *AgosIRP2b*, were detected in all RNA‐sequenced samples. Generally, *AgosIRP2b* exhibited higher expression levels than *AgosIRP2a* in the four morphs of adult aphids. For example, in alate and apterous parthenogenetic females, the expression levels of *AgosIRP2b* were 2.4‐ and 3.2‐fold higher than *AgosIRP2a*, respectively (Figure 6). Both spliced isoforms produce the same mature *IRP*, but *AgosIRP2a* includes an additional enzymatic cleavage that generates an E peptide. The presence of this additional enzymatic cleavage in *AgosIRP2a* suggests that it may produce mature *IRP* at a slower pace compared to AgosIRP2b. The fact that the pathway utilizing *AgosIRP2a* to produce mature IRP was not abandoned indicates that the E peptide may play a role in *A. gossypii*.

Gynoparae, which are produced under short‐day conditions and give rise to sexual females, showed the highest expression levels of both *AgosIRP2a* and *AgosIRP2b* compared to the smallest samples of apterous parthenogenetic females and males. The expression levels of *AgosIRP2a* and *AgosIRP2b* in adult gynoparae were 4.1‐ and 2.4‐fold higher, respectively, than in apterous parthenogenetic females and males (Figure 6). The presence of the female embryo in the adult gynoparae samples suggests that further research is needed to understand the reasons behind the high expression of *AgosIRP2* in gynoparae.

Among the four morphs, gynoparae expressed *AgosIRP*2 at the highest levels, along with the other three widely expressed *AgosIRPs*. All four of the *AgosIRPs* are ubiquitously expressed, with the exception of *AgosIRP*6 having the lowest levels of expression in male. Male was a special morph of aphid that only appeared once a year, and possesses only one X chromosome. In all male developmental stages, *AgosIRP*2a has lower expression levels than *AgosIRP*2b, and this difference is just 0.4‐fold at adult stage (Figure 6). The TPM values of *AgosIRP*2a ranged from 5.6 to 10.8 during the nymph stages while exhibiting highest expression levels at adult stage, with TPM value of 16.5. The expression level of *AgosIRP*2b was decreased with the maturation of male, the TPM value was 57.5 in first instar nymph and 40.5 in mature male (Figure 6).

Apterous viviparous parthenogenetic females were the main morph during aphid outbreaks in the growing season, while alate parthenogenetic females develop when aphid densities increase or the quality of the host plant deteriorates (Brisson, 2010). Apterous females are larger and produce more offspring than alate morphs. Several *A. pisum* insulin‐related peptide genes reported with significant difference in third instar, winged, and wingless nymphs (Guo et al., 2016). In this study, four ubiquitously expressed *AgosIRPs* showed no significant differences between apterous parthenogenetic adult females and alate parthenogenetic adult females. This suggests that *AgosIRPs* may play roles at the stage when winged and wingless nymphs show significant expression level differences.

Transposable element (TE) insertions can affect the up‐ or down‐regulation of immune‐related genes (Rech et al., 2022). In the study, a TE insertion was found in the *AgosIRP3* and *AgosIRP9* genomic regions, which was not present in Hap3 *A. gossypii*. *AgosIRP9* was exclusively found in samples from gynoparae and males, indicating a potential involvement in aphid sexual reproduction stages. However, further analysis revealed that the sexual samples came from an uncharacterized biotype, Hap4 *A. gossypii*, suggesting the need for further study to understand the function of TE insertions in *AgosIRP9* in Hap3 *A. gossypii*.

*AgosIRP*3, which is a ubiquitously expressed gene, had a piggyBac insertion in the CDS region of the Hap3 genome. The TPM value of *AgosIRP*3 in Hap3 was 24.7, higher than in Hap1 (TPM value 12.6), suggesting that this TE insertion is associated with upregulated genes in Hap3 (Figure 6). Additionally, there was one nucleotide difference in the *AgosIRP*3 sequence between Hap1 and Hap3. Interestingly, Hap4 had a similar *AgosIRP3* sequence to Hap1, while Hap3 contained both types of *AgosIRP3* in Japanese aphids but only one form in Chinese aphids.

**FIGURE 7:**
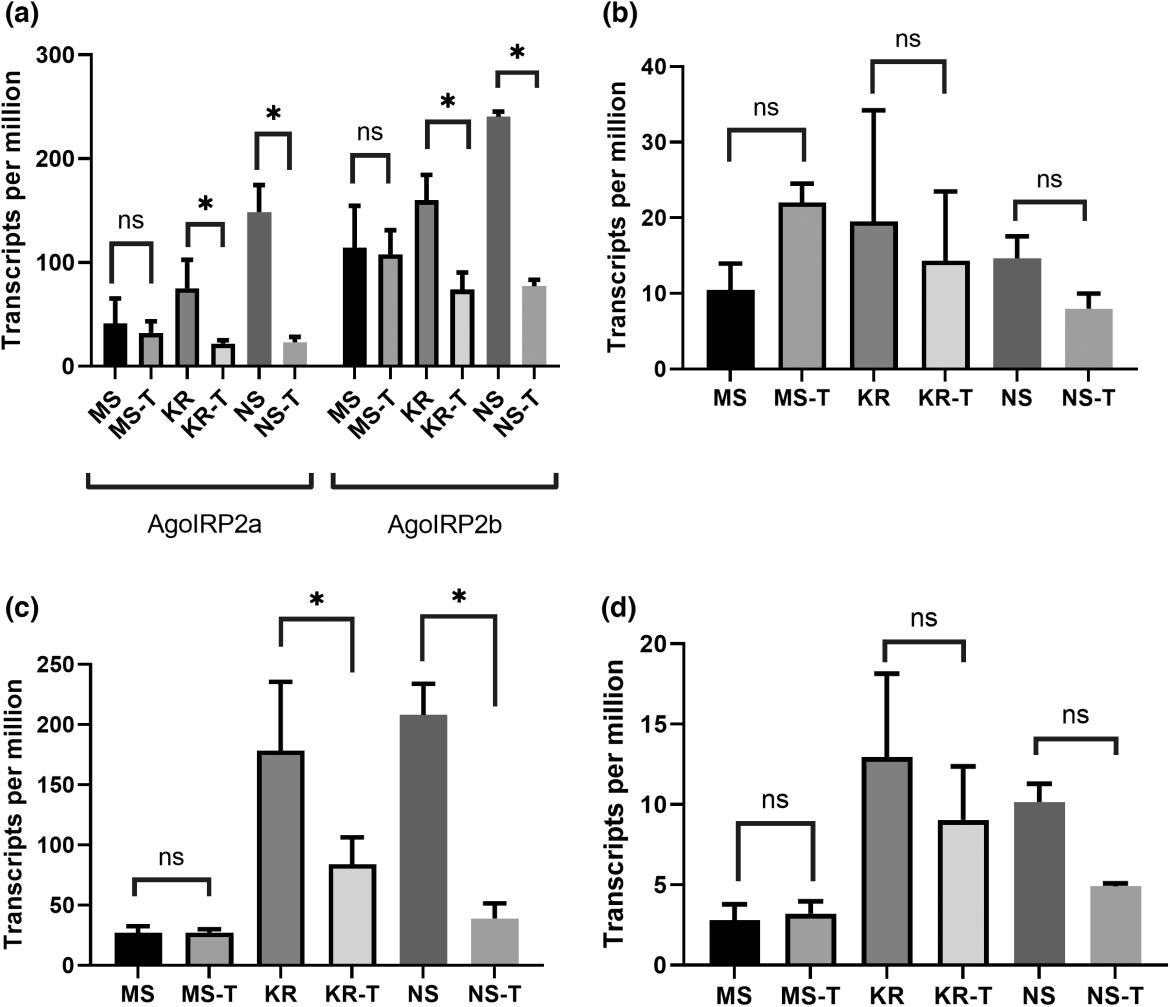
Relative expression quantity of *AgosIRPs* in *Aphis gossypii* treated with pesticide acetamiprid. (a): *AgosIRP2*; (b): *AgosIRP3*; (c): *AgosIRP6*; (d): *AgosIRP7*. The *Y*‐axis was the transcript per million (TPM) value (mean ± standard error of the mean). NS and MS were two susceptible clones, KR was one resistant clone, (T) means with acetamiprid treatment (Hirata et al., 2017). *p < .05, ns, no significant difference, data were analyzed by Mann–Whitney *U* test.

The insecticides have been reported to regulate the expression of insulin‐like peptides in the insects (Chowański et al., 2019; Li et al., 2016). In the study, acetamiprid‐treated samples showed decreased expression levels of *AgosIRPs*, except for *AgosIRP3* and *AgosIRP7* in Miyazaki susceptible (MS) clones. The expression levels of *AgosIRP2* showed variation between insecticide‐resistant and insecticide‐susceptible samples, but there was no correlation between TPM values and insecticide resistance levels. The lowest and highest TPM values were found in two insecticide‐susceptible samples, while the neonicotinoid‐resistant samples exhibited intermediate TPM values. Acetamiprid treatment reduced the expression levels of *AgosIRP2* in all samples (Figure 7).

## 4 CONCLUSIONS

In conclusion, this study sheds light on the diversity and expression patterns of insulin‐related peptides (IRPs) in *A. gossypii*, a polyphagous aphid species. The analysis revealed the presence of multiple IRPs, indicating the specialization of insulin function in different biological processes. The genomic analysis identified evolutionary diversification in the aphid genome, attributed to transposon insertions and alternative splicing events. Furthermore, specific expression profiles of *IRPs* were observed in different morphs and developmental stages of aphids, suggesting their involvement in regulating aphid life cycles, reproductive strategies, host plant preferences, wing polyphenism, and pesticide resistance. These findings provide a valuable resource for further investigations into the evolution of the aphid genome and the functional roles of *IRPs*. Understanding the intricate mechanisms of insulin signaling in aphids can contribute to our knowledge of aphid biology, adaptation, and potential targets for pest management strategies. The comprehensive analysis of *IRPs* in *A. gossypii* presented in this study adds to our understanding of the complex regulatory networks governing aphid physiology and opens up new avenues for future research.

## AUTHOR CONTRIBUTIONS

**Weili Jiang:** Data curation (lead); formal analysis (lead); funding acquisition (lead); investigation (lead); writing – original draft (equal). **Muhammad Nasir:** Writing – original draft (equal); writing – review and editing (equal). **Chenchen Zhao:** Conceptualization (lead); supervision (lead); writing – review and editing (equal).

## CONFLICT OF INTEREST STATEMENT

The authors wish to declare no competing interests.

## Supporting information


Table S1

## Data Availability

The data are available as in the National Center for Biotechnology Information under BioProject accession no. PRJNA936785 and BioSample Accession no. SAMN33368520 and SAMN33368521.
